# The economic impact of the insured patients with severe chronic and acute illnesses: a qualitative approach

**DOI:** 10.3402/gha.v7.22526

**Published:** 2014-10-10

**Authors:** Budi Aji, Shelby Suzanne Yamamoto, Rainer Sauerborn

**Affiliations:** 1 Institute of Public Health, Faculty of Medicine, University of Heidelberg, Germany; 2 School of Public Health, Faculty of Medicine and Health Sciences, Jenderal Soedirman University, Purwokerto, Indonesia

**Keywords:** economic impact, severe illnesses, qualitative study, Indonesia

## Abstract

**Background:**

Little research has focused on the economic hardship among the insured with severe illnesses and high treatment costs, in particular, the consequence of poorer insurance coverage for high-cost illnesses. Therefore, we presented the case for identifying the experiences of insured patients with severe chronic and acute illnesses. This study identified a qualitative understanding of the economic impact of severe chronic and acute illnesses and household strategies to deal with high treatment costs.

**Design:**

Interviews were conducted with 19 insured households of three different health insurance programs with a family member that had been hospitalized for severe chronic or acute illnesses in either Banyumas or Margono Sukarjo hospitals in Banyumas, Central Java, Indonesia. A thematic analysis was applied to guide the interpretation of the data.

**Results:**

Insured households with a family member that had been hospitalized for severe chronic and acute illnesses were greatly affected by the high treatment costs. Four major issues emerged from this qualitative study: insured patients are still burdened with high out-of-pocket payments, households adopt various strategies to cope with the high cost of treatments, households experience financial hardships, and positive and negative perceptions of the insured regarding their health insurance coverage for acute and chronic illnesses.

**Conclusions:**

*Askes* and *Jamsostek* patients faced financial burdens from high cost sharing for hospital amenities, non-covered drugs, and treatments and other indirect costs. Meanwhile, *Jamkesmas* beneficiaries faced no financial burden for related medical services but were rather burdened with indirect costs for the carers. Households relied on internal resources to cover hospital bills as the first strategy, which included the mobilization of savings, sale of assets, and borrowing of money. External support was tapped secondarily and included financial support from extended family members, donations from neighbors and the community, and additional benefits from employers. However, insured households overall had positive perceptions of insurance.

Numerous studies have been conducted to investigate the impact of health insurance in developing countries on household economic resources protection (1–5) . The reason for focusing on this line of research is that health insurance not only ensures access to health services for beneficiaries but also provides financial protection from catastrophic expenditures associated with diseases. The underperformance of health insurance systems in developing countries may lead to an insufficient amount of financial protection among large sections of the population, resulting in high out-of-pocket payments for medical treatments, which, in turn, leads to catastrophic spending and poverty in the long run (6).

Two economic consequences are related to health care utilization, which may lead to household welfare loss: the cost of medical diagnosis and treatment and the loss in income due to labor supply and productivity reduction (7). The role of health insurance is to provide financial protection to eliminate potential loss of household economic resources due to diseases. However, health insurance in many developing countries tends toward only minimum coverage for catastrophic and high-cost treatment of illnesses. In this setting, health insurance will not be able to fully insure against the economic costs of catastrophic illnesses. Moreover, insufficiency of health insurance to alleviate financial burden due to ill-health combined with the costs of labor supply and productivity loss may lead to the household economic hardship.

Illness has different impacts on households, which results in different economic coping strategies and is difficult to generalize across different contexts (8). Previously published works highlight several economic coping strategies of households in response to ill-health. In developing countries, using available cash and savings, selling assets and borrowing, or taking out loans are some of the strategies which households use to cope with the financial burden of health care utilization (8, 9). Another frequent strategy is the labor allocation decision, altered through the substitution of the household economic head by another household member to cope with the loss of income and repay any loans (10).

Little research has focused on the economic hardship among those insured with severe illnesses and high treatment costs, in particular, the consequence of poorer insurance coverage for high-cost illnesses. Gap in empirical knowledge and theoretical understanding of financial hardship among insured and their coping strategies have been also discussed in previous published work by Kinney et al. (11). There is a need for a better understanding of how insured households response to high-cost treatment and their efforts to address the financial burden.

Further, in developing countries, the financial burden of health care utilization is influenced by two factors. First, increasing out-of-pocket spending among the insured is caused by supplier-induced demand. Supplier-induced demand increases insured members’ demands of health services, which leads to overutilization. Providers gain more incentives for this phenomenon, which reinforces this type of behavior. The lack of infrastructure for utilization review to assess the quality of services and the quantity of prescriptions in developing countries is a major contributing factor (12). Second, indirect costs (for example, transportation and loss of work) can contribute substantially to the economic burden of illnesses (13). Therefore, we presented the case for identifying the experiences of insured patients with severe chronic and acute illnesses. This study identified a qualitative understanding of the economic impact of severe chronic and acute illnesses and household strategies to deal with high treatment costs among members of health insurance schemes in Indonesia.

## Indonesian health insurance programs at glance

Indonesia has three different health insurance programs which cover approximately 48% of the population as of 2008, that is, *Jamkesmas, Askes*, and *Jamsostek*. *Jamkesmas* is the largest insurance scheme, which was introduced in early 2005 and covers 74.6 million poor people. *Jamkesmas* offers a generous insurance plan that provides a comprehensive benefits package with a non-contributory premium policy and involves no cost-sharing for all health benefits. Several treatments categorized as luxury health care are not covered by this scheme including cosmetic surgery, dental prostheses, fertility treatment, physical check-ups, and alternative medicine. *Jamkesmas* beneficiaries can access public health care providers all over Indonesia for both outpatient and inpatient services (14).

Established in 1968, *Askes* is the oldest and largest health insurance scheme, covering approximately 16 million civil servants, pensioners of civil servants, and armed forces personnel and their families. This scheme provides comprehensive benefits for both outpatient and inpatient care through a structured health provider mechanism. Benefits are only available through public health care providers in Indonesia. A cost-sharing policy is applied for hospital ward class upgrading, non-formulary drugs, renal dialysis services, transplantation and heart surgery treatments. However, the benefits package excludes procedures such as cosmetic surgery, physical check-ups, alternative medicine, dental prostheses, fertility treatment, and non-basic immunization (15).

A third scheme, *Jamsostek*, was introduced in 1992 to cover formal employees and their dependents in the private sector. *Jamsostek* covers about 4.1 million employees and their spouses and any children below 21 years (up to the 3rd child), or approximately 2% of the population. *Jamsostek* provides a comprehensive benefits package and members are allowed access to both public and private outpatient provider networks. Inpatient care is limited to public hospitals. This scheme does not cover catastrophic health care such as cancer treatment, heart surgery, and renal dialysis (15).

## Methods

To find the appropriate participants or informants, this study employed a purposive sampling approach with criterion sampling strategy. The population from which our sample was drawn included those households insured through *Askes*, *Jamsostek*, or *Jamkesmas* schemes that have faced severe illnesses, which resulted in high medical fees. For example, insured households with members suffering from cancer, cardiovascular diseases, congenital diseases, renal diseases (kidney failure), and illness related to acute accidents fit our inclusion criteria. Interview participants were the head of the household and/or his/her spouse and/or another household member responsible for most of the management of the household as well as those that accompanied insured patients for treatment. The informants were drawn using purposive sampling based on the assumption that the selected participants were persons who understood the phenomena of financial hardship resulting from their catastrophe illnesses.

We also purposively selected hospitals where insured patients had been hospitalized. Given that the benefits package of *Askes*, *Jamsostek*, and *Jamkesmas* for inpatient care only covers public providers, we therefore purposively selected two district and provincial level hospitals in Banyumas, Central Java, Indonesia (i.e. Margono Sukarjo and Banyumas hospitals), which are classified as level B public hospitals. According to the classification system in Indonesia, public hospitals are divided into four classifications, from A to D. Classifications depend on their facilities and capacity to serve patients. Hospitals with advanced facilities and higher capacities are classified as level A, while hospitals with minimal facilities and lower capacities are classified as level D. We selected level B hospitals for the reason that they treat illnesses of greater severity, which increased our potential participant pool. In Banyumas, the highest hospital classification is level B, which is why we selected only the Margono Sukarjo and Banyumas hospitals for this study. The heads of the hospital wards assisted the researcher in selecting insured patients. We recruited 19 participants in both hospitals. A summary of our sampling strategy and sample size is presented in Table 1.

**Table 1:** Qualitative research sampling strategy and sample size

No.	Hospital	Level	Number of beds	Location	Sample size
1	Margono Sukarjo Hospital	B (teaching hospital at provincial level)	507	Banyumas	13
2	Banyumas Hospital	B (teaching hospital at district level)	325	Banyumas	6
Total sample size					19

We employed in-depth interviews with semi-structured and open-ended questions as a method to collect our data. All interviews were conducted in either *Javanese* or *Bahasa*, were recorded digitally, and later transcribed verbatim into English. These strategies were conducted in order to increase the validity and reliability of the study. The interview guide consisted of a brief description of the study, followed by three separate sections of questions and a conclusion. Collected data included demographic information, household health expenditures and strategies to cope with the financial and temporal costs of illnesses, the impact of these financial hardships and high treatment costs as well as informant comments and suggestions. We judged that data saturation had been reached at a point where adequate and quality data had been collected to support the study. This was also the point indicating completion of data collection (16).

Thematic framework analysis was adopted as the main method of data analysis. This step consisted of a detailed analysis to crystallize the concepts, themes and issues contained in the findings. To facilitate this process, we used data coding to ease analyses of larger data files and ensure the accuracy of our analysis. Coding was done according to a main theme and subtheme for each sentence and paragraph. This process was facilitated using a qualitative data analysis software package, MAXQDA 10, which assisted the researcher in sorting and coding the data into conceptual and thematic categories.

Ethical approval was obtained from the ethical committee of the Faculty of Medicine, University of Heidelberg, Germany. Permission to conduct the study was issued by the local government of the district of Banyumas in Indonesia. All participants were recruited voluntarily and provided both verbal and written consent. Anonymity and confidentiality were maintained throughout the process.

## Results

### Characteristic of study participants

Of the 19 participants, seven were *Askes* holders, five were *Jamsostek* holders, and the rest were *Jamkesmas*. Most of them were female (73.7%) and lived outside the district of Banyumas (52.6%). Thirteen participants (68.42%) had a family member who had been hospitalized in Margono Hospital. Most of the participants had a job (68.42%) and nine participants were wives or mothers. Detailed characteristics of the participants are summarized in Table 2.

**Table 2:** Characteristics of study participants

	Frequency	%
Health insurance status		
* Askes*	7	36.8
* Jamsostek*	5	26.4
* Jamkesmas*	7	36.8
Gender		
* *Male	5	26.3
* *Female	14	73.7
Hospital admission		
* *Banyumas Hospital	6	31.6
* *Margono Sukarjo Hospital	13	68.4
Residency		
* *In Banyumas	9	47.4
* *Out of Banyumas	10	52.6
Employment status		
* *Working		
* *Formal and salaried	10	52.6
* *Not salaried	3	15.8
* *Not working	4	21.1
* *Retired	2	10.5
Household relationship		
* *Parents	5	26.3
* *Spouse	8	42.1
* *Child	4	21.1
* *Others	2	10.5

The four major themes that emerged from the in-depth interviews were:

1. Characteristics of out-of-pocket expenditures for insured persons during hospitalization
2. Household strategies to cope with out-of-pocket expenditures due to severe illnesses
3. Financial hardships faced by insured households due to hospitalization
4. Positive and negative perceptions of insured households regarding health insurance schemes

The process of analysis from the thematic framework analysis is provided in Table 3.

**Table 3:** The steps of the thematic framework analysis

Code	Issue discussed	Basic/initial theme	Organizing/developed theme	Global/final theme
Cost-sharing	The amount of the cost-sharing	The amount of expenditures	OOP expenditures related to medically direct costs	Characteristic of out-of-pocket expenditures for insured patient during hospitalization
No cost-sharing	Without cost-sharing			
Covered benefits	The type of covered benefits	Covered benefits scheme		
Uncovered benefit cost	The amount of the negative list benefits costs	Uncovered benefits scheme		
Types of uncovered benefits	The type of the negative list benefits			
Chronic disease	Type of chronic disease	Severity of diseases		
Acute disease	Type of acute disease			
Decision to choose uncovered benefits	Provider convince insured to use branded drugs	Locus of control decision making		
	Learning from past experience to control expenditures			
Ward upgrade	Decision to upgrade ward class		OOP expenditures related to non-medical direct costs	
Hospital LoS	The length of stay in hospital			
Direct cost non-medical	Payment for patient transportation			
	Upgrading facilities			
Costs for accompany	The amount of cost for people who accompanied the patient		OOP expenditures for additional costs	
Meals cost for family	Expenditures for family meals			
Transport cost for family	Expenditures for family transportation			
Using saving for bill	Using family saving for cost-sharing and uncovered benefits	Mobilizing savings	Insured households use their internal resources	Household strategy to cope with out-of-pocket expenditures due to severe illnesses
Selling valuable goods	Selling family assets to cover the bill	Sale of assets		
Borrowing for bill	Decision to borrow some money for the bill	Loans		
Son and daughter support	Support for hospital bill	Family supports	External supports for addressing high OOP expenditures	
	Support for indirect cost			
Parent supports	Financial support from parent			
Relatives support	Financial support for hospital bill			
	Support for daily living patient accompany			
Employer support	Employer providing additional benefit plan	Other benefits plan from employer		
Neighbor support	Neighbor donation	Community support		
Uncertainty solution for a bill	Unclear solution for payments	Many efforts to find solution		
Feel financial burden	Cost-sharing more than income	Influencing monthly income	Economic difficulty influence household income stability	Financial hardships faced by insured households due to hospitalization
	Inability without family support	Income support from family		
	Additional burden			
No feel financial burden	Adequate support from family			
	High level of civil servant grade	Sufficient income		
	Cost-sharing was less than income	Less effect to income		
Having economic impact to household	Decreasing household economic stability	Household economic disruption	High OOP expenditures reduce household assets	
	Affecting household assets	Decreasing household wealth		
No economic impact to household	Having sufficient saving	Less effect to household wealth		
	Scheme provide sufficient coverage			
	Family supports			
Working disruption	Leaving working time	Less working performance	Employment-related issues	
	Reducing working time			
	Reducing salary			
	Influence work performance			
	Inability to work	Employment termination		
	Quit the job			
Grateful for scheme	Positive impression of having scheme	Feeling pleasurable satisfaction	Insured household feel satisfied with the scheme	Positive and negative perceptions of insured households regarding health insurance schemes
	Feeling convenient with the scheme			
	Protecting financial shock	Feeling adequate protection		
	Sufficient benefits coverage			
Past experience of using scheme	Having good experience of having scheme	Good experience using the scheme		
Unhappy with the scheme	Drugs coverage	Insufficient coverage	Insured household feel less satisfied with the scheme	
	Higher cost-sharing			
	Quality of hospital services	Service discrimination		
Another impact	Early discharged from hospital	Other reason to stop the service		

### Characteristics of out-of-pocket expenditures for insured persons during hospitalization

The persistent themes among insured households that had been hospitalized with severe chronic and acute diseases were high cost sharing and non-covered medical costs. Households that were a part of *Askes* and *Jamsostek* insurance schemes experienced substantial cost-sharing for drugs, medical devices, and other non-covered medical expenses. *Askes* and *Jamsostek* holders described the situation as follows:

The total cost for hospital bill was IDR 10,800,000 and Askes covered IDR 7,300,000 so we paid IDR 35,000,000 for the rest. We still had those additional costs […] for some uncovered drugs that were about IDR 930,000. We decided to purchase some branded drugs because sometime the doctor prescribe some drugs that out of Askes list. (A husband, Askes holder, of a wife with kidney failure)

For hydrocephalus surgery we had to buy a pipe [shunt]. The total costs for this surgery was IDR 5,105,000 and Jamsostek only reimbursed IDR 500,000. (A mother, Jamsostek holder, whose son had hydrocephalus)

Members of those with *Jamkesmas* insurance had the opposite of experience of *Askes* and *Jamsostek* members regarding costs. *Jamkesmas* insurance holders are not required to cost share or pay for other medical expenses during hospital stays. For example,

Nothing, we didn't pay anything, thanks God! […] even for ambulance, we only had to provide IDR 500,000 as a bail but then they returned the money. (A wife, Jamkesmas holder, whose husband had a bone fracture)

In this study, hospital amenities greatly influenced the expenditures of insured patients, particularly patients insured through *Askes*. Ward class upgrades and patient transportation by ambulance lead insured patients to spend more in terms of out-of-pocket payments. *Askes* only covers first class wards for the highest grade of civil servant and second and third classes for lower grades. Therefore, many insured patients upgraded to a higher class to access better facilities and coziness reasons. The design of the benefits package of *Askes* allows its members to upgrade the ward class with the consequence of additional charge for the difference in cost of the ward they should receive. For example,

Yes, we upgraded to a higher class ward and we took first class. That's why we had to pay for the difference. It was IDR 50,000 per day. Askes only paid for the second class. (A son, Askes holder, whose mother has tuberculosis and diabetes mellitus)

*Askes* does not cover patient transportation by ambulance, which also increased insured out-of-pocket payments. As one wife said,

We are living in different city and we took ambulance to bring my husband to the hospital and we had to pay IDR 300,000 for that. (A wife, Askes holder, whose husband has diabetes mellitus)

All households whose family members were hospitalized expressed concern about the extra expenditures associated with travel and meal costs in accompanying patients. Even if accommodation was not required because carers were able to stay in the hospital, other costs were still substantial for many households. Money spent for meals and transportation for accompanying family members could be quite high,

[…] for meals and transportation costs that were around IDR 100,000 per day because many relatives accompanied her. We had to provide them meals, cigarettes and miscellaneous. We did not have to pay for the hospital bill but we had to spend a lot of money on accompanying her. (A daughter, Jamkesmas holder, whose mother had bone fracture)

That was about IDR 100,000 per day for meals and others. Totally was around IDR 1,000,000 for a week. (A sister, Jamsostek holder, whose brother had hydrocephalus)

### Household strategies to cope with out-of-pocket expenditures due to severe illnesses

Participants described various coping strategies to deal with the financial costs of treatment including using internal resources and external supports. Internal resources such as saved money, money from the sale of assets, and borrowed money were coping strategies that emerged from the interview with insured households. External support such as assistance from relatives and neighbors were the second most common way to address the financial cost of treatment. Insured households described the use of savings, sale of assets, and taking out of loans to cover medically related costs as follows,

I never spent all my income so I could save some money. And now our daughters have been working to earn their own money. They are not dependent on us again, so I am able to save my monthly income. But now I have to spend my saving for my husband's hospital bill. (A wife, Askes holder, whose husband has stroke, cardiac complications, and kidney failure)

Jamsostek only covered generic drug and lower class of ward. If the costs are more than IDR 3,000,000, Jamsostek will not cover for that. Suppose that Jamsostek covers at least 50% of the total costs that will help us much better. For this case, we had to sell our motor cycle and borrowed some money from our relatives and from my husband office. Any way, we will try our best for our son, whatever! (A mother, Jamsostek holder, whose son has hydrocephalus)

Participants also discussed external provisions that they received from their extended family, neighbors, and even their employers in the form of financial assistance and other types of support. External support significantly reduced potential treatment and additional costs. For example,

Hospital bill was free of charge because we had Jamkesmas. But only for daily living when we were accompanying my husband, for 2 people, me and my daughter. That was around IDR 20,000 per day for meals. However, sometime our relatives visited my husband and they gave us donations then we used it for meals and others. (A wife, Jamkesmas holder, whose husband has bone fracture)

‘Perhutani’ provide us a compensation for hospitalization, because ‘Perhutani’ has ‘Jati Sejahtera’ foundation that compensates their employees and dependents that need hospitalization besides having covered by Askes as well. For my wife hospitalization, I will claim the bill to this foundation to get reimbursement of the ward class upgrading. (A husband, Askes holder, whose wife has kidney failure)

### Financial hardship faced by insured households due to hospitalization

Having a family member hospitalized for a severe chronic disease undergoing costly treatment greatly affected households’ financial resources. Interviewees described the impact of higher cost-sharing on their financial situation. Even after patients were discharged from hospital, households still struggled financially, especially those with substantial cost-sharing expenses. Interviewees were concerned about the impact of the additional medical expenditures on decreasing household income, assets, and employment. Some interviewees described being worried about the loss of income due to higher out-of-pocket payments. These particular circumstances occurred among households insured under *Askes* and *Jamsostek* schemes and were described as follows,

[…] we paid IDR 35,000,000 for the rest […]. If I did not get supports from my daughters, I would feel so difficult to pay the bill. I am a retired civil servant with grade 2 and my monthly income is IDR 1,500,000. My income is only enough for me and my wife […]. Honestly if my daughters did not support us, I would borrow some money or sell our valuable assets to cover the bill. (A husband, Askes holder, whose wife has kidney failure)

Although both of us have incomes, still this situation has influenced our economic conditions. We have bank loan as well. For total cost of hospital bill was IDR 20,000,000, Jamsostek only covered IDR 10,000,000. We are confusing now! (A father, Jamsostek holder, whose son had an accident)

A second impact of the higher out-of-pocket expenses on household economic resources was decreasing household wealth. Participants discussed various household efforts to cope with health care expenditures. Households with higher cost-sharing described selling assets as a way to deal with this particular situation. For example,

[…] totally for the costs of surgery, drugs and class difference, we paid around IDR 10,000,000 […]. That was too much for us because my husband income is around IDR 1,000,000 per month […]. So we sold our motor cycle and borrowed some money from our relatives and my husband office. (A mother, Jamsostek holder, whose son has hydrocephalus)

Another impact of having family members who were hospitalized for severe chronic and high-cost treatment was related to work. The immediate loss of income from loss of work hours was the experience of economic household heads who worked in the private sector. For example,

[…] I had to leave it to accompany my husband and to proceed the claim documents to Jamsostek office. I had left my work for 2 days and 2 over time jobs that was influenced my income because my income depends on working time. For example, I had left a day so my salary will be deducted IDR 50,000. I had left 2 days, it means that my salary will be reduced IDR 100,000. If I leave an over time job so my salary will be deducted IDR 25,000. Normally my salary is IDR 350,000 to 400,000 per weeks. In this case I will receive only IDR 150,000 because I had already left it. (A wife, Jamsostek holder, whose husband has polyps in the colon and rectum)

### Positive and negative perceptions of insured households regarding health insurance schemes

Participants also expressed their perceptions about the services they received under different health insurance schemes. Many participants expressed their relief at having health insurance to assist with incurred expenses from family members who had been hospitalized. In particular, *Jamkesmas* members mentioned the benefit of not having to share costs. *Askes* and *Jamsostek* members also reported fair cost-sharing provisions as part of their health insurance schemes. For example,

All were free of charge […]. We are so lucky to have Jamkesmas. It is really helpful especially for poor people like us. (A wife, Jamkesmas holder, whose husband had an accident)

Total cost was IDR 12,000,000 and we only paid IDR 5,500,000. At least Jamsostek has covered a half of total costs. Jamsostek is somewhat helpful. (A sister, Jamsostek holder, whose brother has hydrocephalus)

Lucky we have Askes. It is really make us comfortable. When we need a doctor we do not have to pay too much. (A mother, Askes holder, whose son had a fracture)

In contrast, some participants expressed less satisfaction with their health insurance while hospitalized. They reported problems with benefits coverage and hospital services. For example,

Jamsostek only covered generic drug and lower class of ward […]. I wish that at least Jamsostek cover more than 50% of the total costs so that would help us much better. (A mother, Jamsostek holder, whose son had hydrocephalus)

[…] the waiting time for a surgery was so long. He had to wait for 15 days, till his wound became infected during delay before surgery […]. My husband did not receive early surgery. We had to wait and wait for a surgery! (A wife, Jamkesmas holder, whose husband had a fracture)

## Discussion

This qualitative study clearly demonstrated the economic burden among insured households from household members hospitalized for severe chronic and acute illnesses. Insured households whose members had been hospitalized due to chronic and acute illnesses were greatly affected by high treatment costs. Insured patients, especially under the *Askes* and *Jamsostek* schemes, experienced higher direct and indirect costs. *Jamkesmas* patients did not report any cost-sharing issues but were less satisfied with the hospital services they received.

Non-covered benefits such as branded drugs, medical instruments, and higher ward classes were the main causes of higher out-of-pocket expenditures among *Askes* and *Jamsostek* insured households. Less adequate benefits schemes for chronic and acute illnesses and supplier-induced demand that encouraged patients to purchase patented rather than generic drugs were a major cause of increased treatment costs among *Askes* and *Jamsostek* members. Providing minimum benefits for all illnesses rather than full coverage for high-cost treatments is a common strategy of social health insurance providers in resource-poor settings (7). This situation leads to a higher amount of cost-sharing, especially after hospitalization due to severe acute and chronic illnesses. Rokx et al. (15) also reported that the level of financial protection regarding out-of-pocket expenditures provided by *Askes* and *Jamsostek* schemes might be limited for members using health services.

The demand for better quality hospital amenities was the second factor that increased out-of-pocket expenditures. Higher ranking civil servants tended to upgrade their ward class from that covered by their basic benefits package. Coziness and quality of service were the main reasons for ward upgrades among the insured. Perception of poor service quality from public hospitals is the reason for this phenomenon (17). As a result, the insured faced additional charges as a consequence of upgrading, which increased the hospital bill that the insured had to pay out of pocket. In some cases, upgrading costs comprised a sizeable share of the overall hospital bill.

Our study highlights an interesting phenomena regarding provider behavior related to branded drug prescriptions. For example, the *Askes* insurance scheme covers 1,646 drugs under its benefits package and also covers severe illnesses. However, physicians sometimes prescribed branded drugs not covered by the scheme instead of generic drugs. Although patients made their own decisions regarding the drugs they chose to take, the common perception of increased efficacy associated with branded drugs encouraged their use over generic drugs. Lack of control by the insurer through claim verification to monitor what services and in what quantities are prescribed also contributed to this phenomenon. Incentives to physicians encouraging the use of branded drugs were an important trigger of higher out-of-pocket payments (18).

Our findings also suggest that there are no extra hospital charges among those insured under the *Jamkesmas* scheme. The current policy for *Jamkesmas* insurance has successfully protected its members against additional treatment expenditures. Under its policy, hospitals have the responsibility of bearing the additional costs from *Jamkesmas* patients if their physicians prescribe any branded drugs or other medical instruments not covered under the scheme. This policy has been effective enough to prevent extra hospital charges. However, this study also found that *Jamkesmas* beneficiaries experienced another financial burden related to the additional costs of transportation, meals, and accommodation for the carer accompanying the patient. These expenditures were substantial for *Jamkesmas* beneficiaries, as well as for *Askes* and *Jamsostek* beneficiaries. Severe acute and chronic illnesses often forced households to seek treatment from more advanced hospitals. Many of these hospitals are at the provincial level and were sometimes quite far from beneficiaries’ residents. This situation leads to additional costs for transportation and accommodation for the carer. Previously published studies found that additional costs such as transportation and accommodation for accompanying persons could be a significant expenditure for insured household and might even exceed 20% of directs costs (8, 19–21) .

This study highlighted a number of strategies employed to cover higher expenditures. Households relied on internal resources to cover hospital bills as a first strategy. External support such as financial support from extended family, donations from neighbors and the community and additional benefits from employers was a second coping strategy. External support from non-kin and community were often used in the case of severe illnesses. Community interaction and involvement has been a longstanding tradition in Indonesian culture. Transactions among households in term of in-kind donations, cash or charity to support a household with a sick person is a common activity in informal networks (8, 9). People preferred to use available cash, mobilize saving, sell assets, receive support from informal networks and community organizations or borrow from a money-lender or credit society to cover the cost of treatment (8, 9, 13, 22, 23). Unfortunately, these coping strategies can be a substantial risk factor for financial calamity and poverty, particularly when patients suffer from severe illnesses, and health insurance coverage is inadequate and a large proportion of a household's economic resources are diverted into treatment (24, 25).

**Fig. 1:**
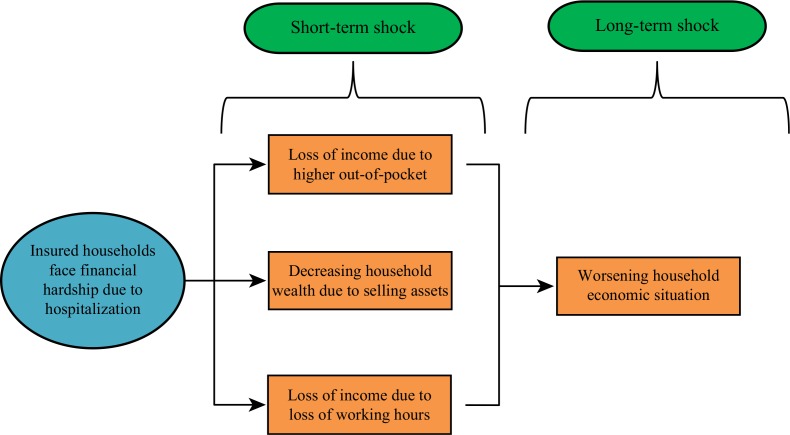
Classification of economic hardship.

Short- and long-term household economic shocks are prevalent even after treatment (see Fig. 1). Devastated income levels, depleted assets, and other work-related issues were the type of short-term impacts on households identified in this study. However, this study was not able to explore longer term impacts such as prolonged household impoverishment or the deepening of household poverty. Although not explored in detail, some participants expressed concern about their worsening household economic situation as a consequence of health care payments. The role of health insurance regarding financial protection was limited when the insured had been hospitalized due to severe illness. Gertler and Gruber (7) also found that health insurance in developing countries tends toward minimum benefits for all illnesses rather than full benefits for rare, high cost, and catastrophic illnesses.

Even though this study did not measure satisfaction among insured households using a specific approach such as the five elements of quality of services (i.e. tangibles, responsiveness, reliability, assurance, and empathy), we were able to capture some perceptions of each health insurance scheme. In general, insured households were positive about their insurance schemes. Insured households perceived that insurance provided more advantages when compared to those that were uninsured, particularly regarding costly medical care. A reduction of the burden of medical expenditures was the main advantage expressed by the participants. Conversely, regarding hospital services, participants reported less satisfaction. For example, *Jamkesmas* beneficiaries mentioned longer waiting times for medical treatment. The unequal distribution and lack of both general and specialized physicians were the major reasons behind long treatment waiting times among *Jamkesmas* patients with chronic and acute illnesses (26, 27). This particular situation contributed to *Jamkesmas* members’ dissatisfaction with the quality of services received under the scheme. Therefore, a government response to this issue to enhance trust in *Jamkesmas* performance is needed.

Our study provided relevant evidence for filling the gap in empirical knowledge and theoretical understanding of financial hardship among insured and their coping strategies. This study has also provided a better understanding of how insured households respond to high-cost treatment and their efforts to address the financial burden. It may also be relevant to policy makers to reform the existing health insurance program in Indonesia to meet the needs of the insured patients who face high-cost treatment. The adequate and affordable health coverage for all insured with serious illnesses should be assured for improving financial protection against the cost of illnesses.

An important limitation of this study is that it was conducted in two hospitals in the district and provincial levels (type B). The public hospital level represents the referral system in Indonesia based on disease severity and complexity. The hospitals are classified hierarchically from the smallest and most basic (type D) to the largest and most technologically advanced (type A), which encounters a higher case mix (28). Given that this study was not conducted in the top national level hospital, the data lacked information on patients with the highest severity. Consequently, access to a higher proportion of people who were likely to experience financial calamity was not possible.

Another limitation of this study is related to the interview process. Obtaining a precise description of the emotional and financial impact was not possible when the participant was not a member of the nuclear family of the insured patient. This particular situation occurred, for example, when the insured patient was an elderly widow or widower who lived alone, and the researcher was only able to interview an extended family member such as a sibling living next door. The extended family member, however, normally assumed responsibility for the care of the insured and was well informed about the financial condition.

## Conclusions

Insured patients with severe illnesses and high-cost treatments need specific policy action to overcome household financial difficulty issues. *Askes* and *Jamsostek* patients faced financial burdens from high cost-sharing for hospital amenities, non-covered drugs and treatments and other indirect costs. Meanwhile, *Jamkesmas* beneficiaries faced no financial burden for related medical services but were rather burdened with indirect costs for the carers. Households relied on internal resources to cover hospital bills as the first strategy, which included the mobilization of savings, sale of assets, and borrowing of money. External support was tapped secondarily and included financial support from extended family members, donations from neighbors and the community and additional benefits from employers. However, insured households overall had positive perceptions of insurance. The reduction of the burden of medical expenditure was the main reason behind insurer satisfaction. In terms of the quality of hospital services, initiating prompt treatment for insurance members with severe acute and chronic illness should be undertaken to further improve satisfaction.
